# Association between Sperm Mitochondrial DNA Copy Number and Concentrations of Urinary Cadmium and Selenium

**DOI:** 10.1007/s12011-023-03868-w

**Published:** 2023-09-27

**Authors:** Cindy Rahman Aisyah, Yuki Mizuno, Momoka Masuda, Teruaki Iwamoto, Kazumitsu Yamasaki, Masahiro Uchida, Fumiko Kariya, Shogo Higaki, Shoko Konishi

**Affiliations:** 1 Department of Human Ecology, The University of Tokyo, Tokyo, Japan; 2 International University of Health and Welfare, Otawara, Japan; 3 Sanno Hospital, Tokyo, Japan; 4 Tsukuba Gakuen Hospital, Tsukuba, Japan; 5 Isotope Science Center, The University of Tokyo, Tokyo, Japan

**Keywords:** Male infertility, Sperm mitochondria, Cadmium, Selenium

## Abstract

Elevated sperm mitochondrial DNA copy number (mtDNAcn) is associated with damage to sperm and poorer measures of semen quality. Exposure to cadmium (Cd) can increase oxidative stress and damage sperm mitochondria. The adverse effects of Cd can potentially be reduced by sufficient selenium (Se). The objective of this study was to examine the associations between sperm mtDNAcn and urinary concentrations of Cd and Se, as well as the Cd/Se molar ratio. Participants were recruited from patients who sought infertility treatment at two hospitals in Japan. Urine and semen specimens and self-administered questionnaires were collected on the day of recruitment. Sperm mtDNAcn was measured in extracted sperm DNA by multiplex real-time qPCR. Urinary Cd and Se concentrations were measured using inductively coupled plasma mass spectrometry, and their molar weights were calculated to obtain the Cd/Se molar ratio. Linear regression was used to estimate associations after adjusting for age, body mass index, smoking, drinking, exercise, varicocele, and hospital of recruitment. Sperm mtDNAcn showed statistically insignificant associations with creatinine-adjusted concentrations of urinary Cd (β = 0.13, 95% CI −0.18, 0.44) and Se (β = −0.09, 95% CI −0.54, 0.35), and Cd/Se molar ratio (β = 0.12, 95% CI −0.13, 0.37). The current study found no evidence of an association between mtDNAcn and urinary concentrations of Cd or Se, or the Cd/Se molar ratio.

**Supplementary Information:**

The online version contains supplementary material available at 10.1007/s12011-023-03868-w.

## Introduction

Infertility is defined as the inability of a couple to conceive within 12 months of unprotected intercourse. From 1990 to 2017, the age-standardized prevalence in 195 countries with high and low socio-demographic indexes has shown an increase, with an estimated annual increase rate of 0.370% in women and 0.291% in men [1]. Infertility can be attributed to both female and male determinants. However, male infertility is more likely to remain undiagnosed than female infertility [2]. Male infertility and its correlates warrant further investigation to lessen the female burden in infertility treatment [3]. Importantly, declining sperm counts were reported in a meta-analysis covering data from 56 countries between 1973 and 2018 [4], while one review study showed the variability of the secular trend of semen quality across populations [5]. These data highlight the urgency of investigating factors relating to semen quality and infertility.

Factors affecting male fertility are mostly evaluated using clinical analyses standardized by the World Health Organization (WHO), including conventional parameters, such as semen volume, sperm motility, morphology, and viability [6, 7]. Another parameter, the total motile sperm count (TMSC), has been introduced as a better indicator of male infertility [8]. Sperm mitochondrial DNA copy number (mtDNAcn) is another biomarker of male infertility associated with fecundity [9]. Sperm mitochondria, located in the midpiece of sperm cells, are the centers of energy production and control apoptosis during spermatogenesis [10, 11]. Sperm mtDNAcn is normally reduced when sperm is produced from spermatogonia; therefore, a higher copy number may indicate abnormal conditions [12]. A systematic meta-analysis study has shown that higher sperm mtDNAcn was associated with lower semen quality parameters [13]. Higher sperm mtDNAcn was also associated with lower embryo quality and a lower pregnancy rate of in vitro fertilization [14] and a higher risk of early pregnancy loss [15].

In the Japanese population, exposure to cadmium (Cd) occurs mainly through diet [16] and adversely affects human health [17, 18]. Cd is a heavy metal and an endocrine-disrupting chemical associated with sperm impairment [18–21]. Cd has been reported to disrupt the electron transport chain in sperm mitochondria, negatively affecting sperm motility and impairing spermatogenesis [22]. Sperm mitochondrial dysfunction may be caused by the inhibition of calcium transport by altering hydrogen ion exchange in the oxidative phosphorylation system [23]. The effect of low Cd exposure on the male reproductive system has prompted concerns about the combined effects of other metals [24].

A number of experimental studies have reported that the physiological effects of Cd exposure can be reduced by selenium (Se), which can protect against Cd-induced oxidative stress in sperm [25, 26]. Several studies have found that increasing the Se dose through supplementation inhibits Cd toxicity in hepatic cells [27] and enhances the metabolism of selenoproteins by altering gut microbiota composition [28]. Se can control redox reactions and reduce the overproduction of reactive oxygen species (ROS) caused by exposure to Cd. The redox reactions occur through the action of Se-containing enzymes, such as glutathione peroxidase (GPx, mainly GPx4), and increased total antioxidant activity, which improves sperm number, motility, and viability [25, 26, 29]. To examine the modification effects of Se on Cd toxicity in an epidemiological study, the ratio of Cd to Se has been used. For example, a study by Al-Saleh et al. showed that the Cd/Se ratio in cord blood and placental tissue was negatively associated with birth weight [30].

Several studies suggest that higher Cd exposure results in decreased sperm quality and higher sperm mtDNAcn. A study using mice and cultured mouse Leydig cells reported that Cd exposure decreased the viability of Leydig cells by increasing ROS levels and reducing mitochondrial membrane potential and ATP production [31]. An increase in the mtDNAcn of the Cd-treated cells was also noted, possibly due to the impairment of mitophagy [31]. Another study reported that prostate cancer patients’ blood Cd was positively associated with blood mtDNAcn [32]. Additionally, a positive association between urinary Cd and a higher proportion of abnormal sperm has been reported [18, 19]. While the protective role of Se against Cd toxicity has been suggested [25, 26], the above-mentioned studies did not examine the protective role of Se against Cd toxicity on sperm quality and sperm mtDNAcn [18, 19]. Therefore, this study aimed to investigate the association between sperm mtDNAcn and urinary concentrations of Cd and Se and the Cd/Se molar ratio.

## Materials and Methods

### Recruitment of Participants

The survey was conducted at hospitals in Tokyo (hereafter referred to as hospital A) and Ibaraki Prefecture (hereafter referred to as hospital B) from September 2019 to March 2020 [33]. We recruited men aged 20–55 years who had not undergone a vasectomy and could provide semen and urine samples on the day of the survey. All participants in both hospitals sought treatment or consultation for infertility.

### General Information of Participants

Physicians specializing in male infertility obtained a full medical history from each study participant and performed a physical examination of each participant [33]. Varicocele was diagnosed based on clinical examination and confirmed by color Doppler analysis. Clinical varicocele severity was graded according to the criteria described by Dubin and Amelar [34]. The participants were categorized into three groups based on their medical records: varicocele (left, right, or both sides), treated, and no varicocele. The participants who underwent varicocelectomy were categorized as treated. Web-based, self-completed questionnaire entries on age, height, weight, smoking status (current, stopped, or never), alcohol drinking frequency (never, once a month, 2–3 times a month, once a week, 2–3 times a week, 4–5 times a week, almost every day), and regular exercise habits (yes or no) were collected. For statistical analyses, both smoking status and drinking frequency were dichotomized, i.e., former and current smokers or those who never smoked, and drinking more than four times a week or not.

### Biological Sample Collection

Semen was collected by masturbation for clinical diagnosis at the recruiting hospitals. The number of days of abstinence was recorded. Urine specimens were collected on the same day as the semen collection. The time of day at urine collection varied among the participants. The participants were not instructed to fast prior to urine or semen collection. Semen samples were stored in 2-mL tubes (AS ONE Co. Ltd., Osaka, Japan), and urine samples were stored in a 15-mL polypropylene conical tube (IWAKI, Asahi Glass Co. Ltd., Japan). Both types of samples were stored at −80 °C in the two hospitals and transferred to the Department of Human Ecology at the University of Tokyo using cold refrigerants for laboratory analysis. No cryoprotection was used for the sample transfer.

### Urinary Cd and Se Concentrations

Urinary Cd and Se concentrations were measured using inductively coupled plasma tandem mass spectrometry (ICP-MS Agilent 8800, Agilent Technologies, Santa Barbara, CA, USA; single MS mode; helium collision mode) at the Isotope Science Center at the University of Tokyo. The urine samples were diluted ten-fold with 0.5% HNO_3_ (ultrapure nitric acid; Kanto Chemical Co., Inc., Tokyo, Japan) and 4% acetic acid (ultrapure acetic acid; Kanto Chemical) [35]. Finally, diluted samples were passed through a 0.45 µm membrane filter (Chubu Scientific. Co., Ltd., Nagoya, Japan).

To prepare working solutions at 1, 5, and 10 µg/kg, a standard multi-element solution (XSTC-622; SPEX, Metuchen, NJ, USA) was used. A standard solution of molybdenum at 100 µg/kg was also included in this measurement to calculate the Cd concentration by removing the effect of molybdenum oxide (MoO) interference using a previously published formula [36]. Yttrium and indium were added to the working solutions and diluted urine samples at 50 µg/kg as internal standard elements for Se and Cd, respectively.

The detection limits for the urinary concentrations of Cd and Se were 0.005 and 0.4 µg/L, respectively. The detection rate was 100% for all targeted elements. Seronorm Trace Elements Urine L-1 and L-2 (SERO AS, Billingstad, Norway) were used for analytical quality assurance. The intra- and inter-assay coefficients of variation (CVs) of urinary Cd and Se measurements are shown in Appendix 1. Two of the five measurements of urinary Cd concentration in our Seronorm Trace Elements Urine L-1 were higher than the reference range, resulting in a higher inter-assay CV (48.3%). This might have been due to the significant influence of uncertainty on the mathematical correction method [36] for the interference of MoO on the observed values when the Cd concentration is extremely low. Appendix 2 shows that the observed urinary Cd concentration in Seronorm Trace Elements Urine L-2 was within the 95% confidence interval (CI) of the analytical uncertainty, whereas the observed urinary Se concentrations in the reference materials were within the 95% CI of the analytical uncertainty.

We used unadjusted, specific gravity, and creatinine adjustments to determine urinary Cd and Se concentrations. The specific gravity of each urine sample was measured using a pocket refractometer (Atago Co., Ltd., Tokyo, Japan). The urinary creatinine concentration was measured by IDEA Consultants, Inc. (Japan) using the enzymatic method [37]. Specific gravity-adjusted urinary Cd and Se concentrations were calculated using the Moore equation [38]:

$$CSG=Craw \times \frac{(SGref-1)}{(SGsam-1)}$$

where CSG is the concentration adjusted by the specific gravity of the sample, Craw is the concentration obtained from the ICP-MS output, SGref is the mean specific gravity of the study population, and SGsam is the specific gravity of the sample. The creatinine-adjusted concentration was determined by dividing the urinary concentration (µg/L) by the creatinine value (g/L).

### Cd/Se Molar Ratio

The Cd to Se molar ratio was calculated as a biomarker to assess the long-term Cd exposure level in relation to Se intake, which attenuates Cd toxicity [30, 39–41]. The molar concentrations (mol/L) of Cd and Se were calculated by dividing the urinary concentrations by their atomic masses (112.41 Cd and 78.96 Se). The molar concentration of Cd was then divided by that of Se to obtain the Cd/Se molar ratio [30]. A ratio greater than 1 indicates more Cd in the urine. Urinary Cd concentration reflects Cd exposure levels in 10–30 years of human life [39, 40], while urinary Se concentration reflects Se exposure levels in the past 3–6 weeks [41].

### Semen Quality Parameters

Semen quality parameters were assessed in each hospital, as described in our previous study [33]. The semen volume was measured using a graduated cylinder with a conical base. Sperm concentration and total motility were quantified using the Sperm Motility and Morphology Analysis System with a computer-assisted semen analyzer (SMAS, DITECT Co., Ltd., Japan). The TMSC was calculated by multiplying the sperm concentration (million per mL), the semen volume (mL per ejaculate), and total motility (%). The reference value used for TMSC was 20 million sperm per ejaculate, which is regarded as a good predictor of a successful pregnancy [8].

### Sperm DNA Extraction

Sperm DNA was extracted at the Department of Human Ecology Laboratory, Graduate School of Medicine, University of Tokyo, according to a previously described method [42]. The isolated dry pellet was resuspended in 500 μL of freshly made RLT buffer (a guanidine-thiocyanate–containing lysis buffer containing guanidine thiocyanate; Qiagen, Hilden, Germany) and tri(2-carboxyethyl) phosphine mixture (TCEP) in the proportion of 1:9. The pellet was homogenized with 0.1 g of stainless steel beads. DNA was extracted from the pellet using a QIAamp DNA Mini Kit (Qiagen Inc., catalog no. 51304). We could not extract DNA from 25 samples due to mistakes in sample processing. Thus, these 25 samples were excluded from the statistical analysis.

### Sperm mtDNAcn Measurement

Sperm mtDNAcn was measured from the extracted sperm DNA using a probe based on multiplex real-time quantitative polymerase chain reaction (qPCR) according to an established protocol [43]. Sperm mtDNAcn was represented as the ratio of the copy number of a mitochondrial region (the minor arc; MinorArc) to that of a nuclear gene (RNaseP). The PCR reaction was carried out on the LightCycler® 96 system (Roche Diagnostics GmbH, Mannheim, Germany) with 8 μL of master mix containing 5 μL of FastStart Essential DNA Probes Master (Roche Diagnostics GmbH, Mannheim, Germany). The DNA sequences of the primers used are listed in Appendix 3.

A pooled sperm DNA sample was used as a standard DNA solution for the calibration curve. All standard curves showed high linearity, with r^2^ > 0.99. Amplification efficiencies were 77–90% and 84–96% for MinorArc and RNaseP, respectively. Quality control samples were run on each plate. All samples were analyzed in triplicate. The intra- and inter-assay coefficients of variation for sperm mtDNAcn measurements were < 5.2% and 2.8%, respectively.

### Statistical Analysis

Statistical analyses were performed using R software version 4.1.2 [44]. Differences between hospitals were tested using the Wilcoxon rank-sum test for age, body mass index (BMI), testicular size (right and left), urinary Cd and Se concentrations, Cd/Se molar ratio, and semen quality parameters. The urinary Cd concentration, urinary Se concentration, Cd/Se molar ratio, sperm concentration, total sperm count, and total motile sperm count were log-transformed. The Fisher’s exact test was used to assess differences in smoking status, frequency of alcohol consumption, exercise, and varicocele status between hospitals. Spearman’s correlation coefficient matrix was calculated to examine the correlation between the following variables: specific gravity, urinary creatinine, urinary Cd and Se concentrations (unadjusted, specific gravity- and creatinine-adjusted), Cd/Se molar ratio, sperm mtDNAcn, semen volume, sperm concentration, total sperm count, total motile sperm count, and total motility. Creatinine-adjusted urinary Cd and Se concentrations were used for comparison with those reported in other studies. A multivariate linear regression model was used to examine the association of urinary Cd and Se concentrations and the urinary Cd/Se molar ratio with sperm mtDNAcn. The covariates included in the linear regression were selected from the possible biological functions that could affect mitochondrial function related to metal exposure, namely age, BMI, frequency of alcohol consumption, smoking status, and exercise [32]. Varicoceles are associated with lower semen quality and higher Cd exposure [45]. Therefore, varicocele status was included as a covariate in this study. Statistical significance was set at *P* < 0.05.

## Results

### General Characteristics of Participants

The complete set of information from 173 of the 198 participants recruited for this study was analyzed. Table 1 shows that the median (interquartile range (IQR)) age and BMI were 36 (33, 42) years and 23 (21, 25) kg/m^2^, respectively. Participants who were recruited at hospital A were significantly older than those who were recruited at hospital B. More than half of the participants never smoked (54%), drank alcohol less than four times per month (54%), and did not exercise regularly (65%). Thirty-five percent of the participants had a one- or two-sided varicocele. The geometric means (geometric standard deviation (SD)) of urinary Cd concentrations in this study were 0.31 µg/L (2.20, unadjusted value), 0.35 µg/L (1.79, specific gravity adjusted), and 0.30 µg/g cre (1.62, creatinine adjusted) (Table 1). Urinary Se concentrations were 32.8 µg/L (1.75, unadjusted value), 36.7 µg/L (1.38, specific gravity adjusted), and 31.9 µg/g cre (1.37, creatinine adjusted). The geometric mean (geometric SD) of the urinary Cd/Se molar ratio for all participants was 6.62 (1.78). Unadjusted and specific gravity-adjusted urinary Se concentrations were significantly higher among the participants recruited at hospital A than among those at hospital B. However, there were no significant differences in urinary Cd concentration or Cd/Se molar ratio between the two hospitals.

**Table 1:** General characteristics of study participants

Variables	Hospital A (*n* = 74)	Hospital B (*n* = 99)	Total (*n* = 173)	*p*-value^d^
Age (years)^a^	39 (34, 44)	35 (32, 39)	36 (33, 42)	** < 0 .001**
BMI (kg/m^2^)^a^	24 (21, 25)	23 (21, 26)	23 (21, 25)	0.615
Smoking status^a^				0.442
Never smoke	43 (58%)	51 (52%)	94 (54%)	
Former or currently smoker	31 (42%)	48 (48%)	79 (46%)	
Frequency of drink alcohol^a^				0.090
< 4 times / month	34 (46%)	59 (60%)	93 (54%)	
≥ 4 times / month	40 (54%)	40 (40%)	80 (46%)	
Exercise^a^				0.750
Yes	25 (34%)	36 (36%)	61 (35%)	
No	50 (66%)	63 (64%)	112 (65%)	
Varicocele^a^				0.081
No varicocele	20 (27%)	37 (37%)	57 (33%)	
Treated varicocele	21 (28%)	34 (34%)	55 (32%)	
Varicocele	33 (45%)	28 (28%)	61 (35%)	
Testicular size				
Right (mL)	20.0 (18.0, 24.0)	23.0 (17.0, 26.0)^e^	19.5 (18.0, 22.0)	**0.003**
Left (mL)	19.0 (16.0, 23.0)	21.5 (17.0, 24.0)^e^	18.0 (16.0, 21.0)	**0.002**
Cd				
Unadjusted (µg/L)^b^	0.36 (1.96)	0.27 (2.33)	0.31 (2.20)	0.071
Specific gravity-adjusted (µg/L)^b^	0.38 (1.72)	0.32 (1.84)	0.35 (1.79)	0.185
Creatinine-adjusted (µg/g cre)^b^	0.30 (1.56)	0.30 (1.67)	0.30 (1.62)	0.824
Se				
Unadjusted (µg/L)^b^	38.3 (1.76)	29.2 (1.70)	32.8 (1.75)	**0.002**
Specific gravity-adjusted (µg/L)^b^	39.8 (1.40)	34.5 (1.35)	36.7 (1.38)	**0.008**
Creatinine-adjusted (µg/g cre)^b^	32.2 (1.41)	31.7 (1.33)	31.9 (1.37)	0.671
Cd/Se^b^^, c^	6.65 (1.73)	6.59 (1.82)	6.62 (1.78)	0.891

*BMI* Body mass index; *Cd* Cadmium; *Se* Selenium

^a^ Median (interquartile range) or n (%)

^b^ Geometric mean (geometric standard deviation)

^c^ Calculated as ((Unadjusted Cd concentration/112.41)/(Unadjusted Se concentration/78.96))

^d^ Wilcoxon rank-sum or Fisher’s exact test. *P* < 0.05 are in bold

^e^ One missing value of both sides of testicular size in hospital B

### Description of Sperm mtDNAcn and Semen Quality Parameters among Participants

**Fig. 1:**
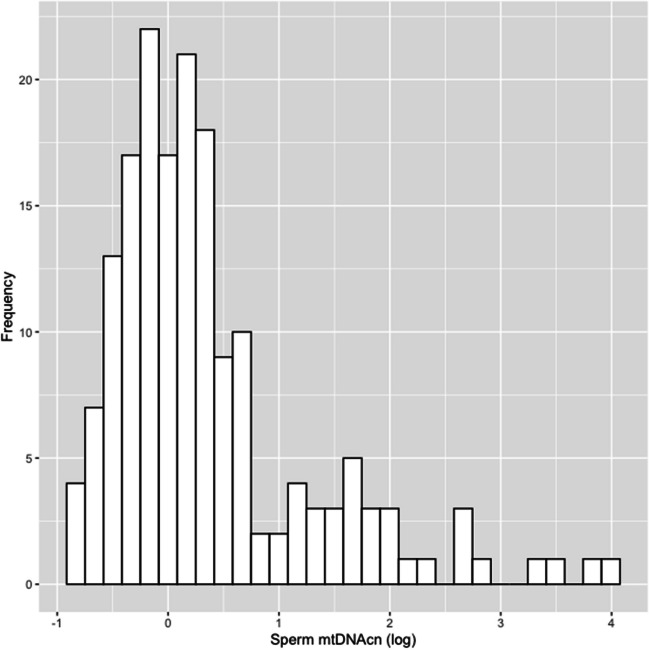
Distribution of sperm mitochondrial DNA copy number (natural logarithm) (*n* = 173)

**Fig. 2:**
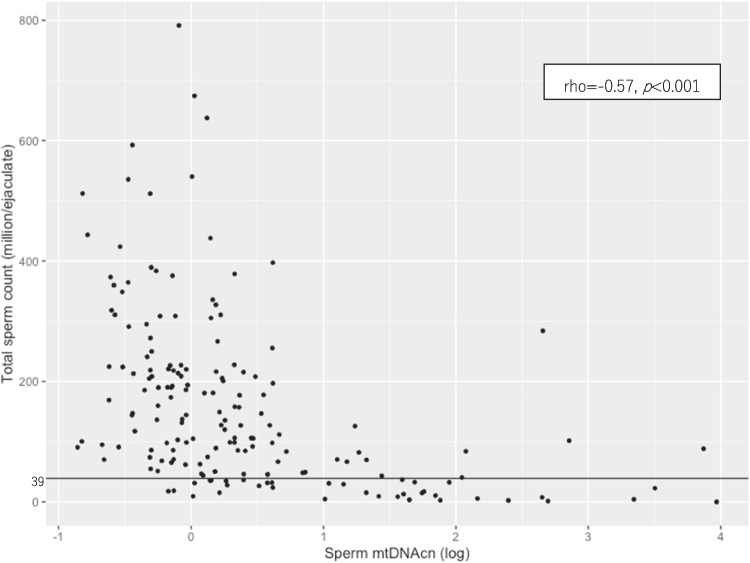
Total sperm count and sperm mtDNAcn in this study (*n* = 173). The horizontal line corresponds to the reference value by the WHO [7]. Spearman’s correlation coefficient and *p*-value are shown

A description of the semen quality of all participants is shown in Table 2. The median mtDNAcn of the sperm was 1.16 (0.79, 1.84). Sperm mtDNAcn values in the two hospitals were similar. Semen volume, sperm concentration, total sperm count, and total motile sperm count were significantly higher among participants recruited at hospital B compared to those recruited at hospital A. The distribution of sperm mtDNAcn was right-skewed (Fig. 1). The scatter plot in Fig. 2 shows a clear negative association between sperm mtDNAcn and the total sperm count. Scatter plots between sperm mtDNAcn and semen volume, sperm concentration, total motility, and total sperm count are shown in Appendix 4.

**Table 2:** Description of sperm mtDNAcn and semen quality parameters of study participants

Variables	Reference	Hospital A (*n* = 74)	Hospital B (*n* = 99)	Total (*n* = 173)	*p*-value^d^
Sperm mtDNAcn^a^	−	1.14 (0.84, 3.06)	1.18 (0.76, 1.61)	1.16 (0.79, 1.84)	0.431
Semen volume (mL/ejaculate)^a, b^	1.4	2.6 (1.6, 3.6)	3.6 (3.0, 4.8)	3.4 (2.3, 4.2)	** < 0.001**
Sperm concentration (million/mL)^a, b^	16	33 (8, 68)	45 (24, 72)	41 (18, 71)	**0.042**
Total sperm count (million/ejaculate)^a, b^	39	69 (27, 167)	177 (87, 253)	120 (49, 219)	** < 0.001**
Total motility (%)^a, b^	42	44 (30, 60)	38 (26, 54)	41 (27, 54)	0.288
Total motile sperm count (million/ejaculate)^a, c^	20	33 (5, 77)	57 (24, 123)	46 (16, 104)	**0.003**

*Sperm mtDNAcn* Sperm mitochondrial DNA copy number

^a^ Median (interquartile range)

^b^ World Health Organization (WHO 2021) lower reference limit at the 5^th^ percentile [7]

^c^ Reference was referred by Hamilton et al. (2015) [8]

^d^ Wilcoxon Rank-Sum test. *P* < 0.05 are in bold

### Correlation between Semen Quality Parameters, Trace Elements, and Sperm mtDNAcn

The Spearman’s correlation matrix (Table 3) showed that sperm mtDNAcn was inversely correlated with all semen quality parameters, except semen volume. Urinary creatinine concentrations and specific gravity were strongly correlated (rho = 0.84). Specific gravity and creatinine levels were negatively correlated with sperm mtDNAcn (rho = 0.16 and 0.18, respectively) and negatively correlated with sperm concentration (rho = −0.20 and −0.26, respectively), total sperm count (rho = −0.22 and −0.26, respectively), and total motile sperm count (rho = −0.23 and −0.26, respectively). The unadjusted urinary concentrations of Cd and Se were negatively correlated with sperm concentration (rho = −0.21 and −0.20), total sperm count (rho = −0.23 and −0.26), and total motile sperm count (rho = −0.26 and −0.25). Sperm mtDNAcn was positively correlated with the unadjusted urinary concentration of Cd (rho = 0.15) and Se (rho = 0.19). However, these correlations became insignificant after urinary concentrations of Cd and Se were adjusted for specific gravity and creatinine concentrations.

**Table 3:** Spearman’s correlation coefficients between urinary Cd and Se concentrations, urinary Cd/Se molar ratio, sperm mtDNAcn, and semen quality parameters (*n* = 173)

Variables	[a]	[b]	[c]	[d]	[e]	[f]	[g]	[h]
Specific gravity [a]	1.00							
Creatinine [b]	0.84***	1.00						
Unadjusted-Cd [c]	0.63***	0.75***	1.00					
Unadjusted-Se [d]	0.81***	0.85***	0.66***	1.00				
SG-Cd [e]	0.04	0.26***	0.75***	0.18*	1.00			
SG-Se [f]	−0.01	0.18*	0.18*	0.48***	0.29***	1.00		
Cre-Cd [g]	−0.03	−0.02	0.60***	0.02	0.85***	0.08	1.00	
Cre-Se [h]	−0.15*	−0.34***	−0.25***	0.15*	−0.20**	0.57***	0.03	1.00
Cd/Se [i]	0.04	0.15*	0.65***	−0.09	0.84***	−0.22**	0.83***	−0.49***
Sperm mtDNAcn [j]	0.16*	0.18*	0.15*	0.19*	0.11	0.11	0.08	−0.02
SV [k]	−0.09	−0.06	−0.10	−0.16*	−0.07	−0.16*	−0.06	−0.14
SC [l]	−0.20**	−0.26***	−0.21**	−0.20**	−0.09	−0.03	−0.04	0.09
TSC [m]	−0.22**	−0.26***	−0.23**	−0.26***	−0.10	−0.11	−0.06	0.00
TMSC [n]	−0.23**	−0.26***	−0.26***	−0.25***	−0.12	−0.06	−0.09	0.04
TM [o]	−0.08	−0.06	−0.14	−0.05	−0.08	0.08	−0.12	0.06

Variables	[i]	[j]	[k]	[l]	[m]	[n]	[o]
Specific gravity [a]							
Creatinine [b]							
Unadjusted-Cd [c]							
Unadjusted-Se [d]							
SG-Cd [e]							
SG-Se [f]							
Cre-Cd [g]							
Cre-Se [h]							
Cd/Se [i]	1.00						
Sperm mtDNAcn [j]	0.03	1.00					
SV [k]	0.03	−0.05	1.00				
SC [l]	−0.07	−0.60***	−0.11	1.00			
TSC [m]	−0.03	−0.57***	0.33***	0.87***	1.00		
TMSC [n]	−0.08	−0.61***	0.26***	0.84***	0.92***	1.00	
TM [o]	−0.12	−0.37***	0.01	0.42***	0.36***	0.66***	1.00

[a] Urinary specific gravity; [b] Urinary creatinine; [c] Unadjusted Cd concentration (µg/L); [d] Unadjusted Se concentration (µg/L); [e] SG-Cd, urinary Cd concentration adjusted by specific gravity (µg/L); [f] SG-Se, urinary Se concentration adjusted by specific gravity (µg/L); [g] Cre-Cd, urinary Cd concentration adjusted by creatinine (µg/g cre); [h] Cre-Se, urinary Se concentration adjusted by creatinine (µg/g cre); [i] Cd/Se molar ratio (calculated as urinary [Unadjusted Cd ÷ 112.41] ÷ [Unadjusted Se ÷ 78.96]); [j] sperm mitochondrial DNA copy number; [k] semen volume (mL); [l] sperm concentration (million/mL); [m] total sperm count (million/ejaculate); [n] total motile sperm count (million/ejaculate); [o] total motility (%)

Statistically significant values are indicated by asterisks: *P* < 0.001 (***), *P* < 0.01(**), and *P* < 0.05 (*)

### Associations of Sperm mtDNAcn with Urinary Concentrations of Cd and Se

In multivariate linear regression analysis, the associations between sperm mtDNAcn and the unadjusted urinary concentrations of Cd (β = 0.15, 95% CI −0.03, 0.33) and Se (β = 0.19, 95% CI −0.07, 0.45) were positive but not statistically significant (Table 4). A positive but statistically insignificant relationship was also found between the specific gravity-adjusted urinary Cd concentrations and sperm mtDNAcn (β = 0.18, 95% CI −0.07, 0.42). There were no clear associations between specific gravity-adjusted Se concentrations and sperm mtDNAcn (β = 0.21, 95% CI −0.23, 0.65). Associations between the creatinine-adjusted urinary concentrations of Cd and Se with sperm mtDNAcn were statistically insignificant (β = 0.13, 95% CI −0.18, 0.44 and β = −0.09, 95% CI −0.54, 0.35, respectively). Furthermore, the Cd/Se molar ratio did not show a statistically significant association with sperm mtDNAcn (β = 0.12, 95% CI −0.13, 0.38). Among the predictors in all models, participants with varicocele were more likely to have significantly higher sperm mtDNAcn compared to those who had no varicocele (Table 4).

**Table 4:** Multivariate linear regression model result on the associations of urinary Cd and Se concentrations or urinary Cd/Se molar ratio with sperm mtDNAcn (*n* = 173)

Predictors	Model 1 Beta and 95% confidence interval (β, 95%CI)	Model 2 Beta and 95% confidence interval (β, 95%CI)	Model 3 Beta and 95% confidence interval (β, 95%CI)	Model 4 Beta and 95% confidence interval (β, 95%CI)	Model 5 Beta and 95% confidence interval (β, 95%CI)	Model 6 Beta and 95% confidence interval (β, 95%CI)	Model 7 Beta and 95% confidence interval (β, 95%CI)
Unadjusted Cd^a^	0.15 (−0.03, 0.33)						
Specific gravity-adjusted Cd^b^		0.18 (−0.07, 0.42)					
Creatinine-adjusted Cd^c^			0.13 (−0.18, 0.44)				
Unajdjusted Se^a^				0.19 (−0.07, 0.45)			
Specific gravity-adjusted Se^b^					0.21 (−0.23, 0.65)		
Creatinine-adjusted Se^c^						−0.09 (−0.54, 0.35)	
Cd/Se^d^							0.12 (−0.13, 0.37)
Age (years)	−0.01 (−0.04, 0.01)	−0.01 (−0.04, 0.01)	−0.01 (−0.04, 0.01)	−0.01 (−0.03, 0.02)	−0.01 (−0.03, 0.02)	−0.01 (−0.03, 0.02)	−0.01 (−0.04, 0.01)
BMI (kg/m^2^)	0.01 (−0.03, 0.05)	0.01 (−0.03, 0.05)	0.01 (−0.03, 0.05)	0.01 (−0.03, 0.05)	0.01 (−0.03, 0.05)	0.01 (−0.03, 0.05)	0.01 (−0.03, 0.05)
Current or former smoking	−0.13 (−0.42, 0.15)	−0.16 (−0.45, 0.12)	−0.16 (−0.45, 0.13)	−0.10 (−0.38, 0.19)	−0.13 (−0.41, 0.16)	−0.13 (−0.41, 0.16)	−0.15 (−0.44, 0.14)
Drinking alcohol > 4 times per month)	0.12 (−0.08, 0.32)	0.14 (−0.06, 0.33)	0.14 (−0.06, 0.34)	0.12 (−0.08, 0.32)	0.14 (−0.06, 0.34)	0.15 (−0.05, 0.35)	0.14 (−0.06, 0.34)
No exercise	0.24 (−0.06, 0.53)	0.24 (−0.05, 0.54)	0.24 (−0.06, 0.54)	0.23 (−0.06, 0.53)	0.24 (−0.06, 0.53)	0.22 (−0.08, 0.52)	0.23 (−0.06, 0.53)
Varicocele status (ref. = no varicocele)							
Treated	0.32 (−0.02, 0.65)	0.31 (−0.03, 0.65)	0.31 (−0.03, 0.65)	0.33 (−0.01, 0.67)	0.33 (−0.01, 0.67)	0.31 (−0.03, 0.65)	0.30 (−0.04, 0.64)
Varicocele	**0.36 (0.02, 0.69)**	**0.36 (0.03, 0.70)**	**0.37 (0.03, 0.70)**	**0.38 (0.05, 0.72)**	**0.39 (0.05, 0.73)**	**0.37 (0.03, 0.71)**	**0.36 (0.02, 0.69)**
Hospital (ref. = hospital B)	−0.11 (−0.40, 0.19)	−0.12 (−0.42, 0.17)	−0.14 (−0.44, 0.15)	−0.08 (−0.38, 0.22)	−0.10 (−0.40, 0.21)	−0.14 (−0.43, 0.16)	−0.15 (−0.44, 0.15)
R^2^	0.083	0.079	0.071	0.079	0.072	0.068	0.072

*BMI* Body mass index; *CI* 95% confidence interval; *mtDNAcn* Mitochondrial DNA copy number; *Cd* Cadmium; *Se* Selenium; *Cd/Se* Urinary cadmium and selenium molar ratio, calculated as ((Unadjusted Cd concentration/112.41)/(Unadjusted Se concentration/78.96))

Models 1–6 analyzed sperm mtDNAcn association with Cd and Se in unadjusted, specific gravity-, and creatinine-adjusted values, respectively. Model 7 analyzed sperm mtDNAcn with the Cd/Se molar ratio. All models were adjusted for age, BMI, smoking status, alcohol consumption frequency, exercise, varicocele status, and hospital stay. *P *< 0.05 are in bold

^a^ log-transformed, unadjusted concentration (µg/L)

^b^ log-transformed, concentration adjusted by specific gravity (µg/L)

^c^ log-transformed, concentration adjusted by creatinine (µg/g creatinine)

^d^ log-transformed, Cd/Se molar ratio

## Discussion

The current study hypothesized that higher exposure to Cd, as monitored by urinary concentration of Cd, would manifest as increases in sperm mtDNAcn, which may be suppressed by a higher intake of Se, as measured by urinary concentration of Se. However, our multivariate analyses of urinary Cd and Se concentrations, as well as the Cd/Se molar ratio, showed insignificant associations with sperm mtDNAcn, regardless of which adjustment of urinary concentration was applied.

Regarding the correlation between sperm mtDNAcn and semen quality parameters, a higher sperm mtDNAcn in the current study was associated with low sperm concentration, low total sperm count, low total motile sperm count, and low total motility, which is consistent with a previous study [13]. Sperm mtDNAcn is a biomarker of sperm mitochondrial function [11]. It has been hypothesized that the elimination of sperm mtDNA during spermatogenesis is important for maintaining effective sperm energy production by reducing ROS-mediated damage to the mtDNA [12]. If the amount of ROS is excessive, it may also alter mitochondrial intermembrane metabolism, making sperm mitochondria more susceptible to oxidative stress and lowering sperm quality [10–12].

This study observed a negative correlation between urinary Cd and Se and semen quality parameters. In a pilot study by Toshima et al., a higher Cd concentration was associated with lower semen quality parameters in the general population of Japan [18]. The Cd effect on lower sperm quality was observed even at relatively lower concentrations [45]. Cd can induce the exchange of hydrogen and essential ions in the electron transport chain, causing damage and reducing cell viability [20, 22]. Cd is positively associated with higher sperm DNA fragmentation and acrosome reactions, which correlate with cell damage and sperm abnormalities [46, 47]. Another study using mice revealed a possible mechanism by which exposure to Cd could damage testes by decreasing testicular testosterone, reducing levels of superoxide dismutase, and affecting lipid peroxidation [48]. In contrast, two previous studies in China did not observe any correlations between urinary Cd concentrations and semen quality parameters [49, 50]. The results of this study were also inconsistent with a study in China in which lower urinary Se was associated with lower sperm count in infertile patients [50]. Other studies in China showed no association between semen quality parameters and urinary Se in healthy donors [49] or seminal plasma Se in infertile patients [51]. The positive associations of Se concentration in seminal plasma with sperm concentration and total sperm count were mediated by lower sperm mtDNAcn [52]. The possibility that another element, such as iodine, is involved in the regulation of selenoproteins in male reproductive function might be worthy of consideration [53, 54]. Differences in study design, such as variations in the number of sampling points per person, may have contributed, at least partially, to the inconsistent results across studies.

The Cd/Se molar ratio was calculated to assess the relationship between Cd exposure and Se intake rather than relying solely on a single element’s concentration in the urine [30]. We observed no statistically significant association between the Cd/Se molar ratio and any of the semen quality parameters or sperm mtDNAcn. A recent epidemiological study examined the potential effects of the metal mixture, including Cd and Se, and showed a negative, but insignificant, association with semen quality parameters [24]. While specific gravity- or creatinine-adjusted concentrations of Cd and Se were not associated with sperm mtDNAcn, unadjusted Cd and Se concentrations showed a significantly negative correlation with sperm mtDNAcn. The differences in the associations could be related to the positive correlation of urinary specific gravity and creatinine concentration with sperm mtDNAcn. However, there is currently no established biological explanation for such associations of urinary specific gravity and creatinine with sperm mtDNAcn. A negative association was observed between seminal creatinine concentrations and sperm count in patients with intracytoplasmic sperm injection [55], but it is unknown whether urinary creatinine concentration reflects seminal creatinine concentration. Thus, the findings of this previous study [55] do not explain the associations observed in our study.

The comparison of urinary Cd and Se concentrations in the present study with those in previous studies used creatinine-adjusted values, which are shown as geometric means and medians (Table 5). The geometric mean (SD) of urinary Cd concentration in this study was 0.30 (1.62) µg/g creatinine and the median urinary Cd (IQR) was 0.31 (0.22, 0.42) µg/g creatinine. The mean urinary Cd concentration observed in this study was comparable to the value reported in a national survey in the USA (geometric mean 0.34 µg/g creatinine) [56]; however, it was lower than the concentration found in Japanese adults living in non-polluted areas (geometric mean 1.80 µg/g creatinine) [17], as well as the concentrations obtained in two studies in Wuhan, China [50, 57], which had geometric means of 0.60 and 0.86 µg/g creatinine, respectively. The urinary Cd concentration in this study was slightly higher than the general population in the USA [46] and young healthy sperm donors in Chongqing, China [58], with a median of 0.16 and 0.24 µg/g creatinine, respectively. For urinary Se concentration, the geometric mean (SD) was 31.9 (1.37) µg/g creatinine, and the median (IQR) was 31.2 (25.6, 37.7) µg/g creatinine. The urinary Se concentration in this study was similar to that in healthy young Japanese individuals with a median of 30.9 µg/g creatinine [59], and the general population in the USA with a geometric mean of 29.0 µg/g creatinine [46]. The urinary Se concentration observed in this study was higher than that of studies in China where Se deficiency is prevalent; within healthy males with a median (IQR) was 6.27 (2.51, 11.2) µg/g creatinine [58], as well as the geometric mean of urinary Se concentrations in other two studies, was 8.92 µg/g creatinine [50] and 14.0 µg/g creatinine [57].

**Table 5:** Comparison of urinary Cd and Se concentrations (µg/g creatinine) in the present and previous studies

Element	Study information			Concentration (µg/g creatinine)
	Reference	Location	Participants (n)	
Cd	This study	Japan (this study)	173	0.30 (1.62)^a^ 0.31 (0.22, 0.42)^b^
	A national survey, NHAES III (Menke et al. 2008) [56]	USA	1262	0.34 (0.003, 4.22)^b^
	Non-polluted area (Suwazono et al. 2015) [17]	Japan	1067	1.80 (2.4)^a^
	Infertile patients (Zeng et al. 2015) [50]	China	394	0.60^a^
	General population (Wan et al. 2019) [57]	China	746	0.86^a^
	General population (Branch et al. 2021) [46]	USA	413	0.16 (0.1)^a^
	Healthy males (Chai et al. 2022) [58]	China	703	0.24 (0.04, 0.61)^b^
Se	This study	Japan (this study)	173	31.9 (1.37)^a^ 31.2 (25.6, 37.7)^b^
	Infertile patients (Zeng et al. 2015)[50]	China	394	8.92^a^
	General population (Wan et al. 2019) [57]	China	746	14.0^a^
	General population (Branch et al. 2021) [46]	USA	413	29.0 (19.34)^a^
	Healthy young adults (Fuse et al. 2022) [59]	Japan	10	30.9 (24.0, 39.8)^b^
	Healthy males (Chai et al. 2022) [58]	China	703	6.27 (2.51, 11.2)^b^

^a^Geometric Mean (Geometric Standard Deviation); most studies did not report the geometric standard deviation

^b^Median (IQR range)

We observed that participants with an untreated varicocele had a higher sperm mtDNAcn compared to those without varicocele. Varicoceles are associated with lower semen quality resulting from sperm DNA damage and increased oxidation–reduction potential in the normozoospermic population [60]. A significant reduction in sperm mtDNAcn was reported four months after microsurgical varicocelectomy [61]. Therefore, varicocele treatment could lessen mitochondrial inactivity and lower sperm mtDNAcn [60–62].

The strength of this study lies in its consideration of the mixed effect of Cd and Se associated with sperm mtDNAcn rather than a single effect. However, there are some limitations that should be mentioned. Our results may not be representative of the general population because the data were obtained from volunteers who visited the hospital for infertility treatment. Moreover, the Cd/Se molar ratio was calculated using the concentration of a one-time spot urine specimen. In addition, other toxic substances that may contribute to higher mtDNAcn were not analyzed. Biomarkers for oxidative stress, which have been hypothesized to mediate the link between Cd exposure and mtDNAcn, were also not assessed. Finally, we did not measure Cd and Se concentrations in semen samples, which may have a more direct correlation with sperm mtDNAcn compared to their urinary concentrations.

## Conclusions

A higher sperm mtDNAcn was related to lower sperm quality parameters, as previously described. This study did not find any evidence of an association between urinary Cd or Se and sperm mtDNAcn. We found that individuals with untreated varicocele and higher creatinine concentrations tended to have higher sperm mtDNAcn. These findings may enrich our knowledge of semen quality parameters and their association with environmental exposure.

### Supplementary Information

Below is the link to the electronic supplementary material.

Supplementary file1 (DOCX 213 kb)

## Data Availability

The datasets generated during and/or analyzed during the current study are not publicly available to protect participants’ personal information but are available from the corresponding author upon reasonable request.
