# Supplementary material for: Association between Sperm Mitochondrial DNA Copy Number and Concentrations of Urinary Cadmium and Selenium
Source: Biol Trace Elem Res. 2023 Sep 27;202(6):2488–500. doi: 10.1007/s12011-023-03868-w (PMC11052814; doi:10.1007/s12011-023-03868-w)
Supplement: Supplementary file 1 — Supplementary file1 (DOCX 213 kb) [file 12011_2023_3868_MOESM1_ESM.docx]

**APPENDICES**

**Association between Sperm Mitochondrial DNA Copy Number and Concentrations of Urinary Cadmium and Selenium**

Cindy Rahman Aisyah^1^, Yuki Mizuno^1^, Momoka Masuda^1^, Teruaki Iwamoto^2,3^, Kazumitsu Yamasaki^3,4^,

Masahiro Uchida^4^, Fumiko Kariya^1^, Shogo Higaki^5^, Shoko Konishi^1^

^1^ Department of Human Ecology, The University of Tokyo, Japan, ^2^ International University of Health and Welfare, ^3^ Sanno Hospital, ^4^ Tsukuba Gakuen Hospital, ^5^ Isotope Science Center, The University of Tokyo

16-digit ORCIDs

Cindy Rahman Aisyah (0000-0003-3915-9804)

Yuki Mizuno (0000-0002-7383-4706)

Momoka Masuda (0009-0009-7522-2664)

Kazumitsu Yamasaki (0000-0002-8414-2393)

Shogo Higaki (0000-0002-5098-6475)

Shoko Konishi (0000-0002-7282-0863)

**Corresponding author**

Correspondence to Shoko Konishi (email: [moe@humeco.m.u-tokyo.ac.jp](mailto:moe@humeco.m.u-tokyo.ac.jp))

**Appendix 1** Detection limit and inter- and intra-assay CVs (%) of urinary Cd and Se measurements

| **Elements** | **m/z** | **Detection limit (µg/L)^a^** | **Intra-assay CV (%)^b^** | **Inter-assay CV (%)^b^** |
| --- | --- | --- | --- | --- |
| Cadmium (Cd) | 111 | 0.005 | 6.19, 1.09 | 48.3, 1.58 |
| Selenium (Se) | 78 | 0.4 | 11.4, 1.31 | 3.44, 2.43 |
| ^a^Detection limit obtained by calculating the mean of all detection limits in each measurement (n = 5).  ^b^The values of intra- and inter-assay CVs (%) are shown for each of Seronorm Trace Elements Urine L-1 and L-2, respectively. | | | | |

**Appendix 2** Ranges of observed and reference concentrations of Cd and Se in Seronorm Trace Elements Urine L-1 and L-2 (µg/L)

| Elements | Seronorm L-1 | | Seronorm L-2 | |
| --- | --- | --- | --- | --- |
|  | Observed (µg/L) | Reference (µg/L) *^a^* | Observed (µg/L) | Reference (µg/L)^a^ |
| Cadmium (Cd) | 0.06 – 0.17 | 0.05 – 0.07 | 4.41 – 4.65 | 3.7 – 5.6 |
| Selenium (Se) | 11.3 – 12.4 | 8.4 – 12.6 | 61.9 – 66.7 | 51.9 – 80.6 |
| *^a^* Reference for analytical uncertainty was the 95% confidence interval, as specified in the Seronorm manufacturer’s manual. | | | | |

**Appendix 3** DNA sequences used for quantification of MinorArc and RNaseP by multiplex real-time quantitative polymerase chain reaction (qPCR)

| Primer Sequences | |
| --- | --- |
| Forward MinorArc | 5’-CTA AAT AGC CCA CAC GTT CCC-3’ |
| Reverse MinorArc | 5’-AGA GCT CCC GTG AGT GGT TA-3’ |
| Forward RNaseP | 5’-AGA TTT GGA CCT GCG AGC G-3’ |
| Reverse RNaseP | 5’-GAG CGG CTG TCT CCA CAA GT-3’ |
| Probe Sequences | |
| MinorArc | FAM-CAT CAC GAT GGA TCA CAG GT-BHQ1-MGB |
| RNaseP | Cy-TTC TGA CCT GAA GGC GGC TCT GCG CG-BHQ1-MGB |

MinorArc was targeted to quantify the control region in human mtDNA where no deletions have been reported.

The cycling conditions were set as follows: activation for 10 min at 95°C, followed by 40 cycles of 95°C for 15 s, 55°C for 15 s, and 60°C for 1 min.

**Appendix 4**. Scatter plots of sperm mtDNAcn versus (a) semen volume, (b) sperm concentration, (c) total motile sperm count, and (d) total motility

| (a)  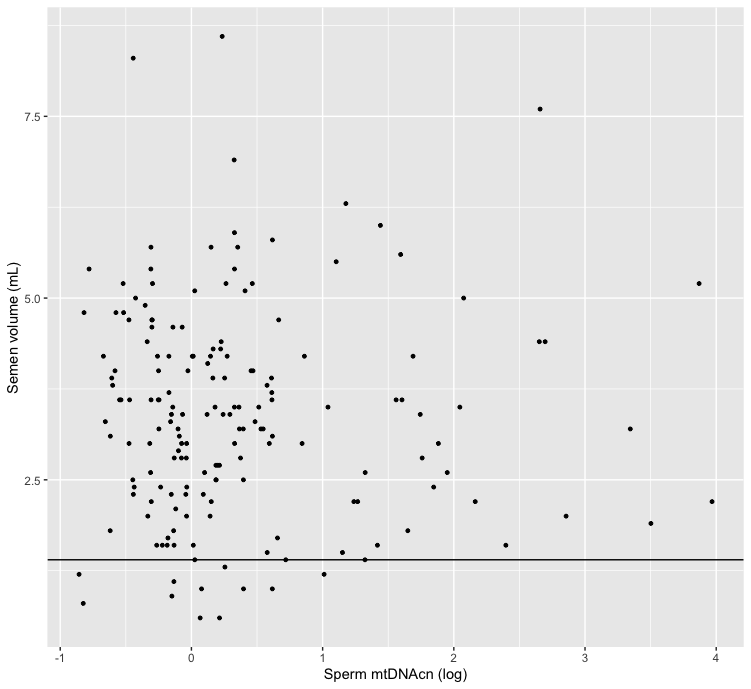  rho=-0.05, *p*=0.49  1.4 | (b)  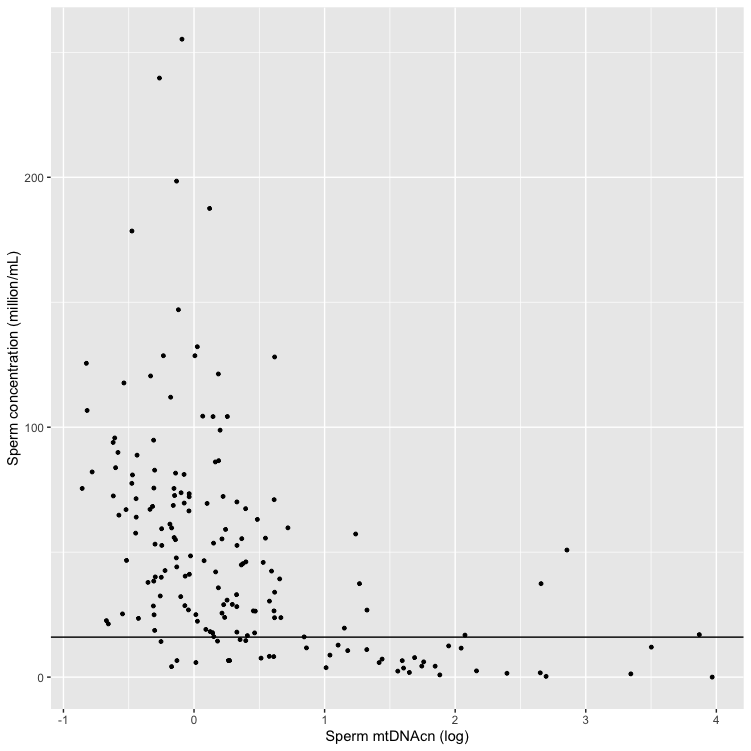  rho=-0.6, *p*<0.001  16 |
| --- | --- |
| (c)  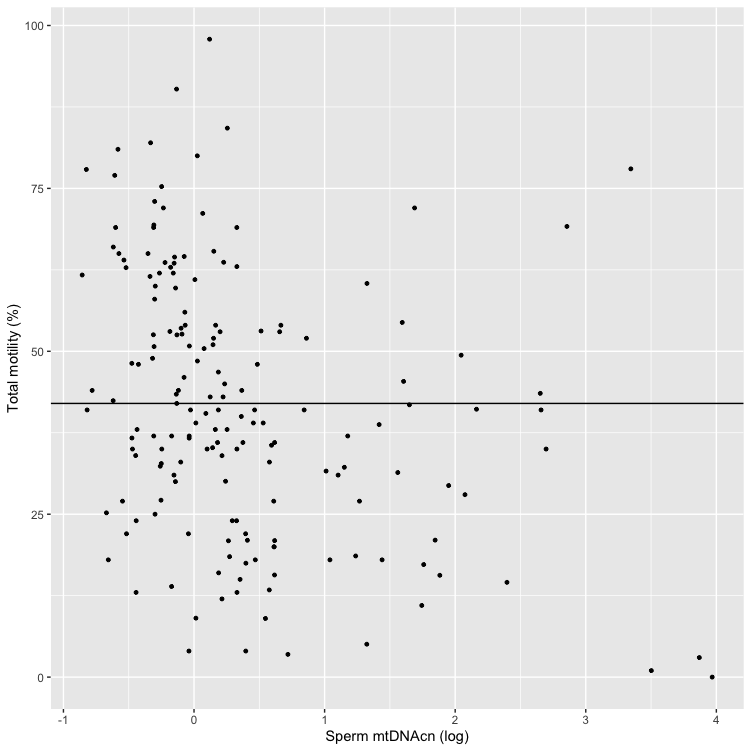  rho=-0.37, *p*<0.001  42 | (d)  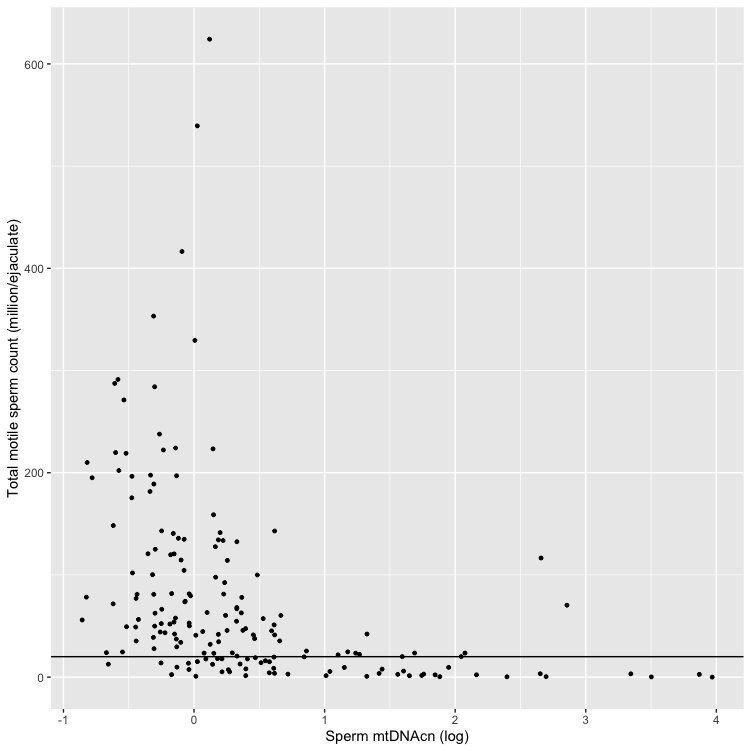  rho=-0.61, *p*<0.001  20 |

Sperm mtDNAcn = sperm mitochondrial DNA copy number

Note: The horizontal line corresponds to the reference values by the WHO [7] for (a) semen volume, (b) sperm concentration, and (c) total motility. The horizontal line in (d) total motile sperm count corresponds to the reference of the study by Hamilton, 2015 [8]. Spearman’s correlation coefficients and *p*-values are shown.
